# Supplementary material for: Zika virus prM protein contains cholesterol binding motifs required for virus entry and assembly
Source: Nat Commun. 2023 Nov 13;14:7344. doi: 10.1038/s41467-023-42985-x (PMC10643666; doi:10.1038/s41467-023-42985-x)
Supplement: Supplementary file 1 — Supplementary Information [file 41467_2023_42985_MOESM1_ESM.pdf]

Supplementary information

Zika virus prM protein contains cholesterol binding motifs required for virus entry and assembly

Sarah Goellner, Giray Enkavi, Vibhu Prasad, Solène Denolly, Sungmin Eu, Giulia Mizzon, Leander Witte, Waldemar Kulig, Zina M. Uckeley, Teresa M. Lavacca, Uta Haselmann, Pierre-Yves Lozach, Britta Brügger, Ilpo Vattulainen, Ralf Bartenschlager

Corresponding author: ralf.bartenschlager@med.uni-heidelberg.de

This PDF contains supplementary figures, figure legends, and tables.

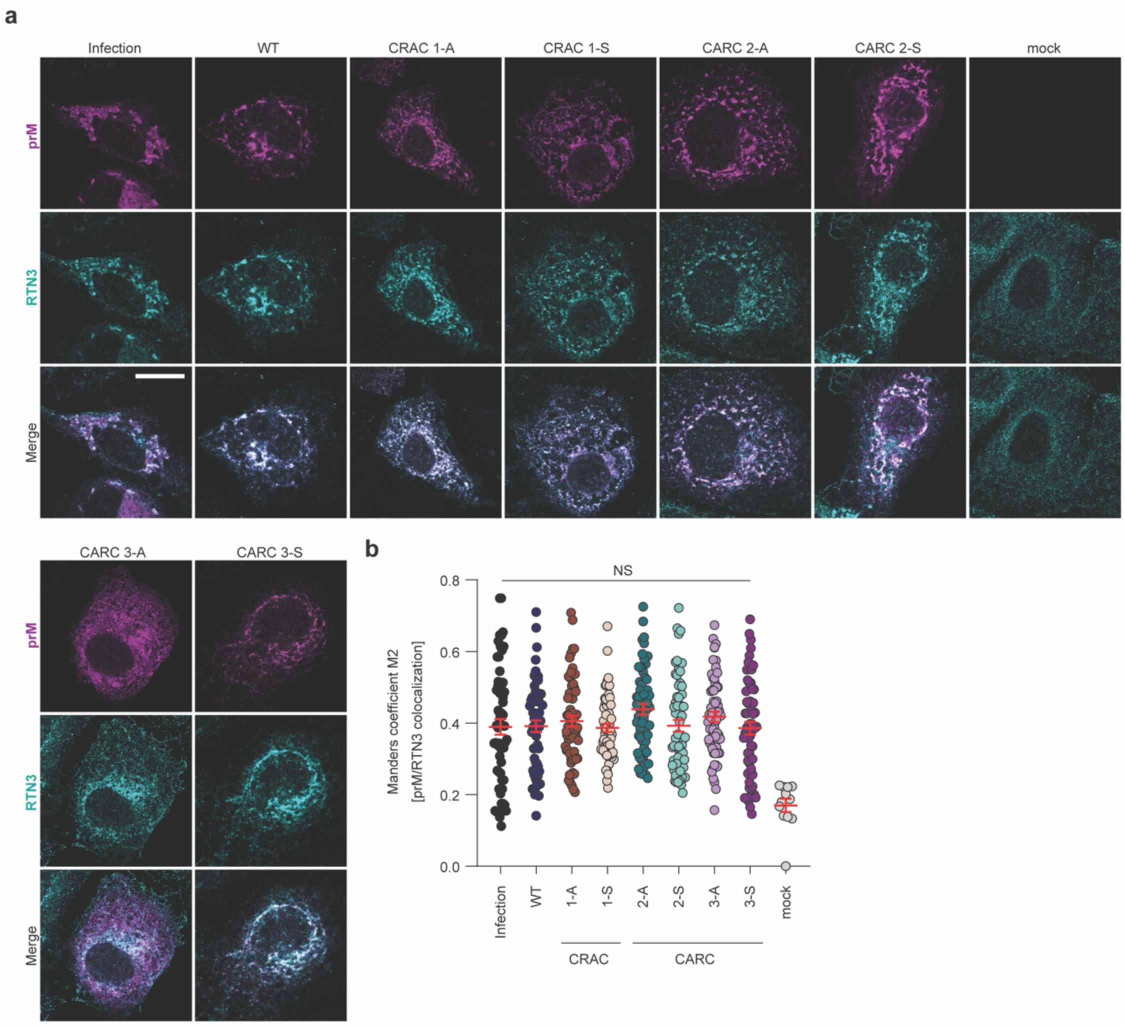

Supplementary Figure 1. Subcellular localization of prM-HA proteins
(a) Huh7-Lunet T7 cells were seeded onto glass coverslips and were either infected with ZIKV H/PF/2013 (MOI=5) or transfected with pTM prM-HA WT and mutant constructs specified. After 18 and 24 hours transfected and infected cells, respectively, were PFA fixed and subjected to immunofluorescence analysis for prM (magenta) and reticulon-3 (RTN3; cyan), a well-established ER marker. Scale bar: 20 μm. **(b)** Quantitative analysis of colocalization of prM and RTN3 in infected and transfected cells. M2 co-occurrence coefficients (fraction of prM overlapping with RTN3) were estimated employing the Fiji JACoP plugin. Data are mean ± SEM from 60 cells. n=3 independent experiments. Kruskal-Wallis test. NS not significant.

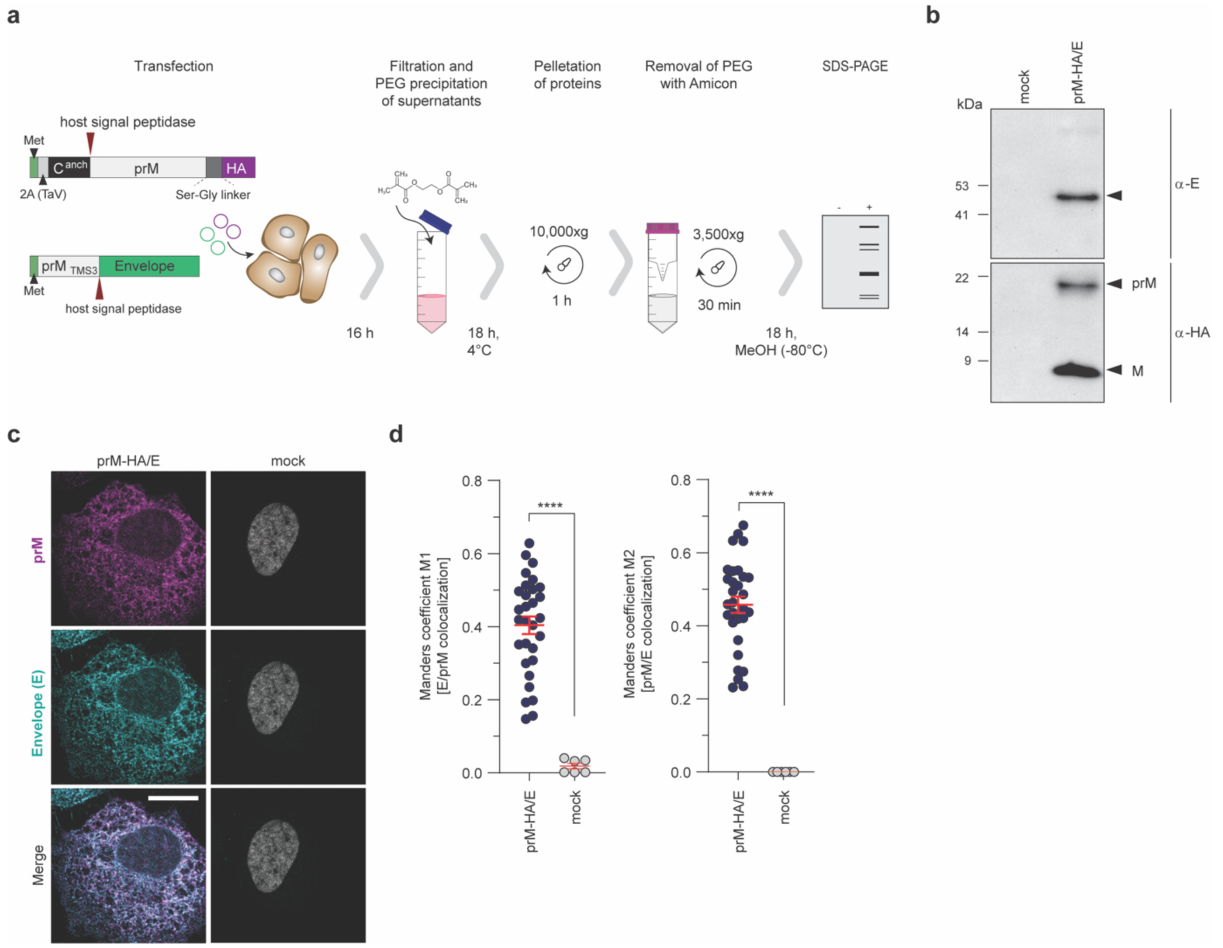

Supplementary Figure 2. Co-expression of HA-tagged prM and E gives rise to ZIKV subviral particles.

(a) Experimental set up. Huh7-Lunet T7 cells were transfected with pTM-derived prM-HA and E encoding constructs or mock transfected. After 18 hours, cell culture supernatants were collected, and secreted proteins contained therein were precipitated using polyethylenglycol (PEG). On the following day, proteins were pelleted via centrifugation and washed twice with PBS using a 100K Amicon filtration device. Retained proteins were precipitated using methanol and reconstituted in equal volumes. Proteins were analyzed by western blot. **(b)** Secreted E and prM-HA proteins were detected by immunoblotting using E- and HA-specific antibodies. A representative result is shown (n=2). Molecular sizes of proteins are indicated on the left (in kilodalton; kDa). **(c)** Huh7-Lunet T7 cells were seeded onto glass coverslips and co-transfected with pTM derived prM-HA and E encoding constructs. After 18 hours cells were PFA fixed and subjected to immunofluorescence analysis to detect prM (magenta) and E (cyan). Scale bar: 20 μm. **(d)** Colocalization analysis of prM-HA and E in transfected cells. M1 (fraction of E overlapping with prM-HA) and M2 (fraction of prM overlapping with E) co-occurrence coefficients were determined employing the Fiji JACoP plugin. Data are mean ± SEM (n=2). Two-tailed t test with Welch's correction, ****P<0.0001.

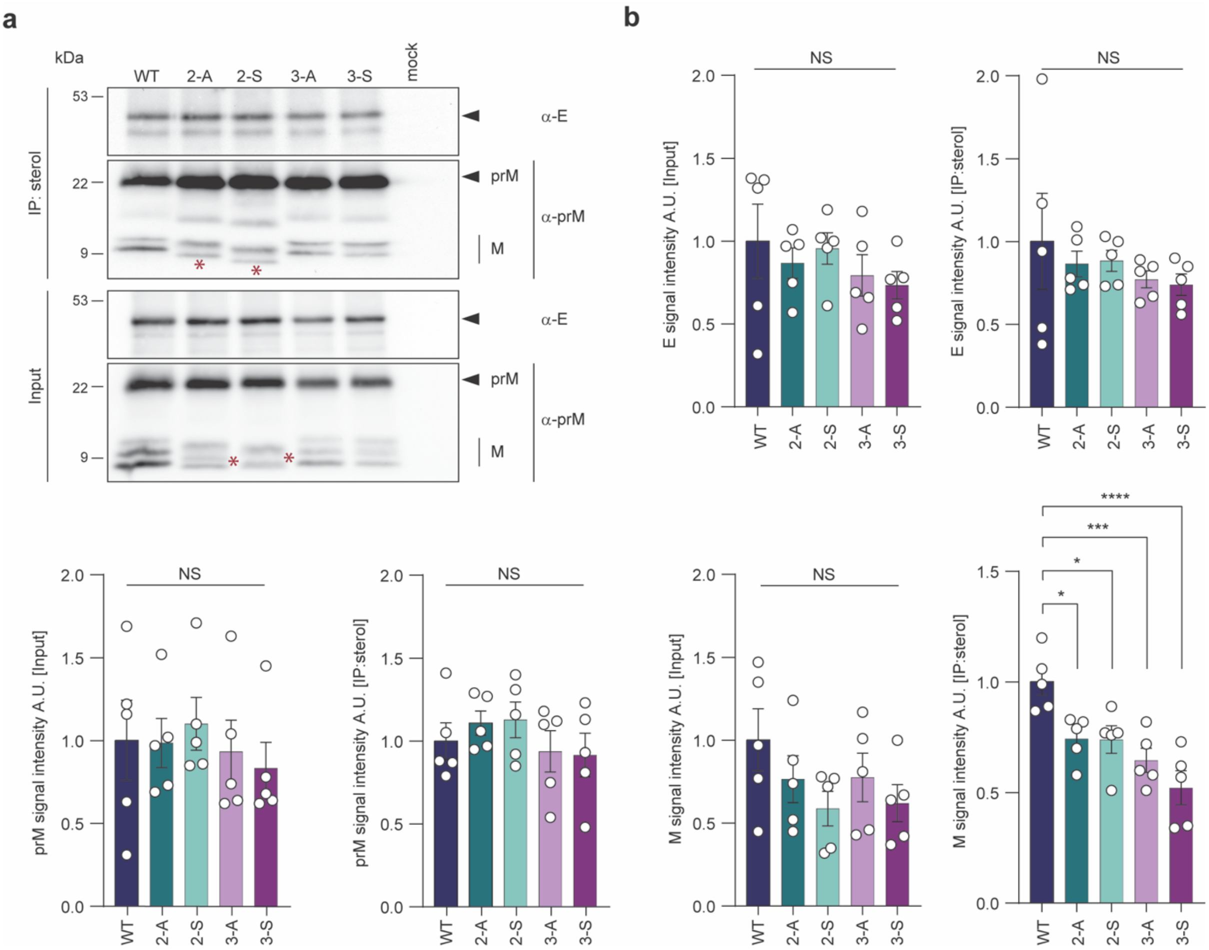

Supplementary Figure 3. Mutations within TMS2 and TMS3 of prM reduced cross-linking ability to cholesterol.
(a) Huh7-Lunet T7 cells were transfected with pTM prM/E ZIKV VLP WT or mutant constructs for 16 hours. Cells were fed for one hour with 10 μM PAC-cholesterol probe alongside with 5 μM of the proteasome inhibitor MG132. After crosslinking by UV irradiation, clarified cell lysates were subjected to biotinylation via click chemistry. Lipid–protein complexes were captured using neutravidin conjugated resin beads and analyzed by western blot using ZIKV prM- and E-specific antibodies. Asterisks mark differences of M-protein patterns observed for the CARC2 mutants relative to WT proteins. Molecular weights are indicated on the left (in kilodalton; kDa). A representative western blot is shown (n=5). **(b)** Relative abundance of ZIKV E, prM and M proteins was calculated by densitometry of western blots (n=5). Protein amounts were normalized via Bradford assay prior to click chemistry. Data are mean ± SEM (n=5). One-way ANOVA with Dunnett's test, *P<0.05, ***P<0.001, ****P<0.0001. (WT vs 2-A, P=0.0129; WT vs 2-S, P=0.0116; WT vs 3-A, P=0.0009). NS not significant.

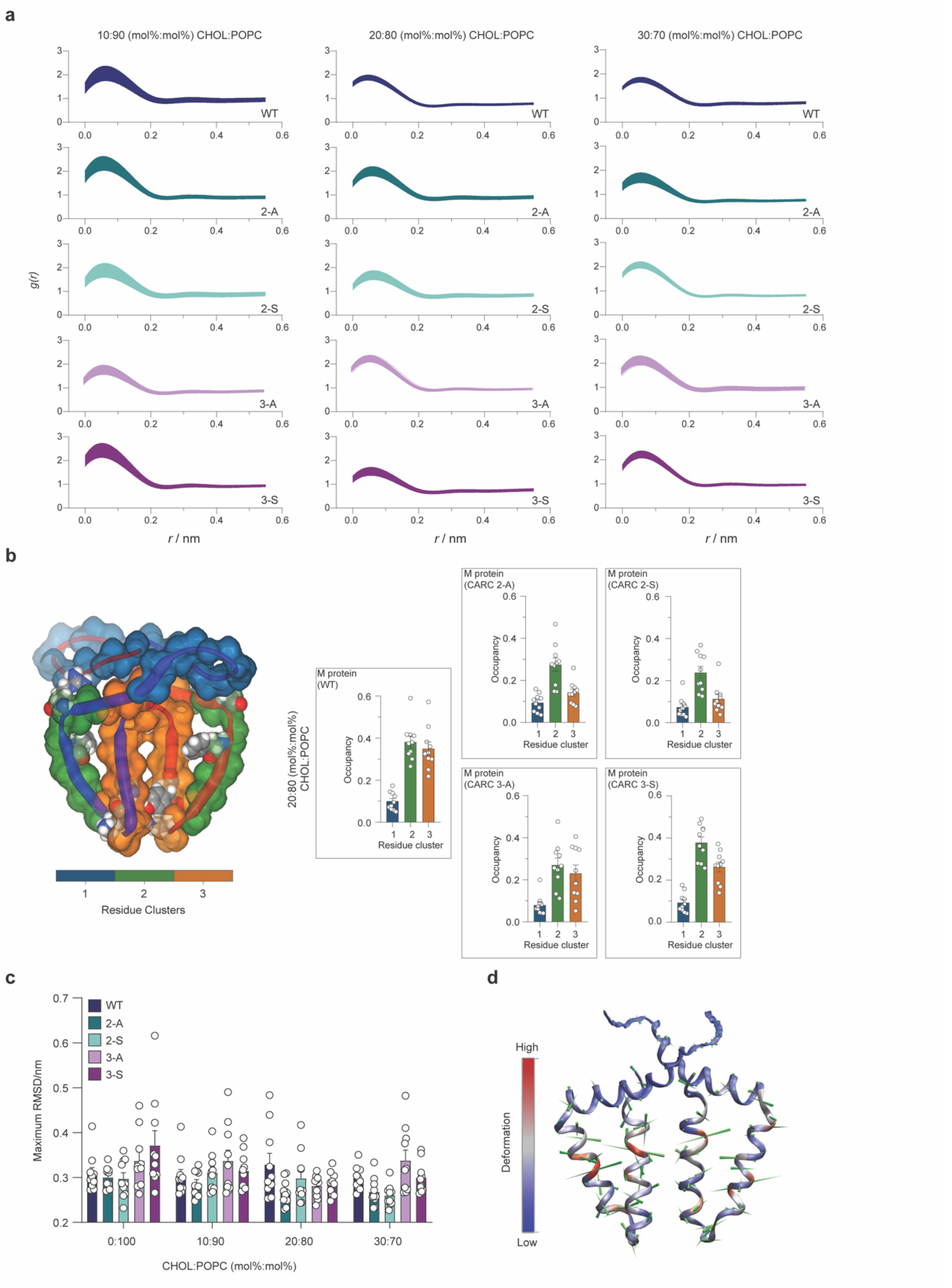

Supplementary Figure 4. Atomistic molecular dynamics simulations

(a) Two-dimensional radial distribution functions (g(r) along radial in-plane distance r) of cholesterol with respect to the protein surface. Band thicknesses indicate the

standard error. **(b)** Residue clusters that form distinct cholesterol binding sites are highlighted on the protein structure (left). The occupancy of each site for WT and mutant M proteins are shown for 20:80 (mol%:mol%) CHOL:POPC bilayer (right). CARC2 and CARC3 mutations reduce cholesterol binding to sites 2 and 3. Error bars indicate SEM. **(c)** The mean maximum Root Mean Square Deviation (RMSD) of the transmembrane C_α atoms of M is shown as bar plots. The maximum RMSD does not exceed 0.4 nm. Averages are taken over ten independent repeats. Error bars indicate SEM. **(d)** A porcupine plot for the first Linear Discriminant Analysis (LDA) component indicating the mode of the conformational change with increasing cholesterol concentration. The deformations of protein C_α atoms were scaled by a factor of 10 for visual clarity and are shown as green cones. The protein backbone is also coloured by the scale of deformation.

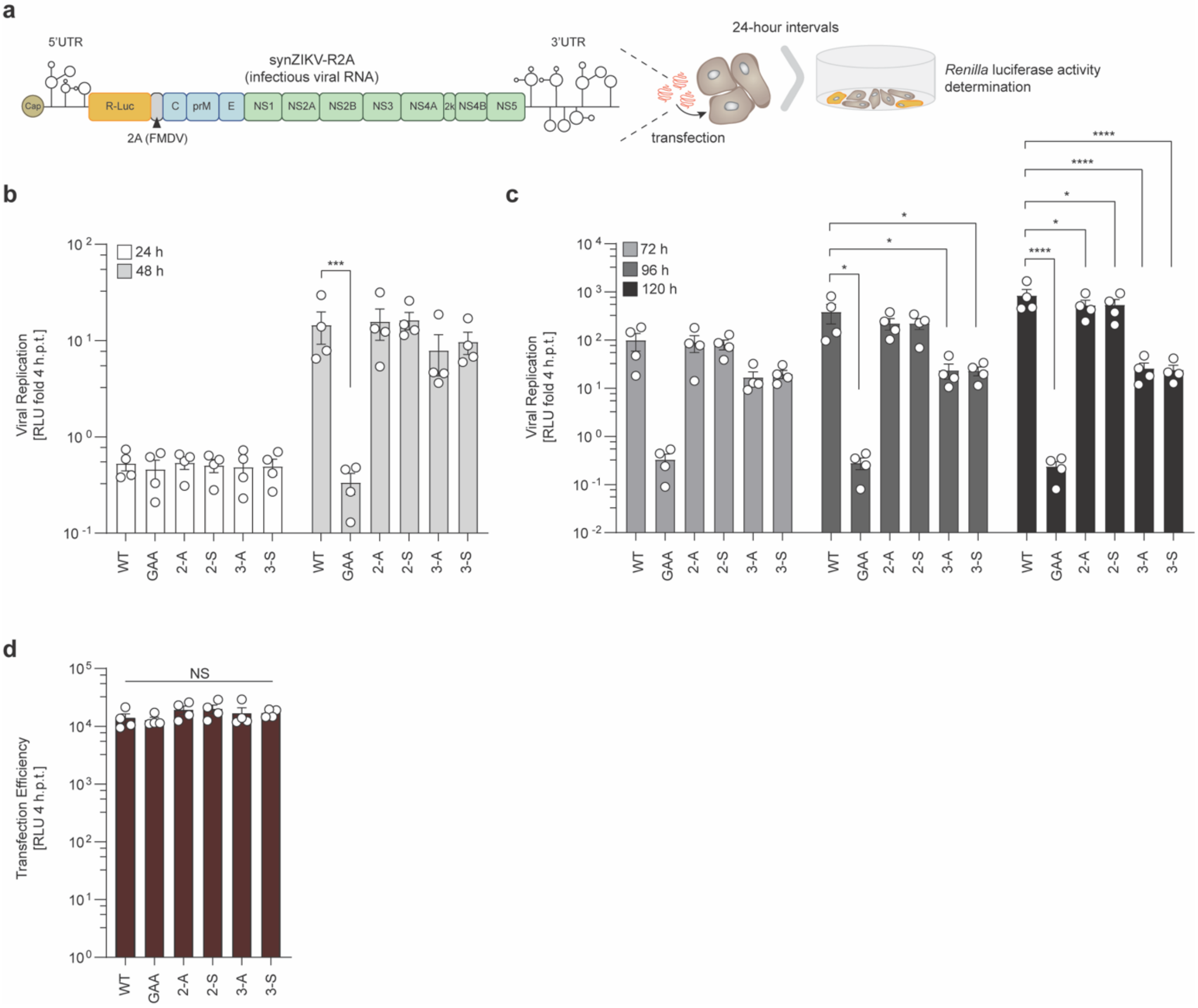

Supplementary Figure 5. Mutations targeting the CARC3 motif within the M-domain of the prM protein reduce viral replication.
(a) Schematic representation of the synZIKV-R2A-H/PF/2013 reporter virus genome and viral replication assay. The 2A coding sequence from the food and mouth disease virus (FMDV) mediates the separation of the *Renilla* luciferase from the ZIKV polyproteins. In transfected cells, *Renilla* luciferase activity reflects the level of viral replication; values determined at 4 hours after transfection indicate transfection efficiency and are used for normalization. **(b, c)** Huh7-Lunet cells were transfected with *in vitro* transcripts of the synZIKV-R2A genome (H/PF/2013 isolate) and *Renilla* luciferase activity was measured in 24-hour intervals over a period of 120 hours. Relative light units (RLUs) normalized to the respective 4-hour value are blotted. Data are mean ± SEM (n=4). Two-way ANOVA with Fisher's LSD test, ***P<0.001. ((b) WT vs GAA, P=0.0008; (c) 96h WT vs GAA, P=0.0066; WT vs 3-A, P=0.0104; WT vs 3-S, P=0.0103; (c) 120h WT vs 2-A, P=0.0244; WT vs 2-S, P=0.0292). **(d)** Absolute RLU values 4 h.p.t. reflecting transfection efficiency. Data are mean ± SEM (n=4). One-way ANOVA with Dunnett's test. NS not significant. Data in panels (b) - (d) are from the same experiments.

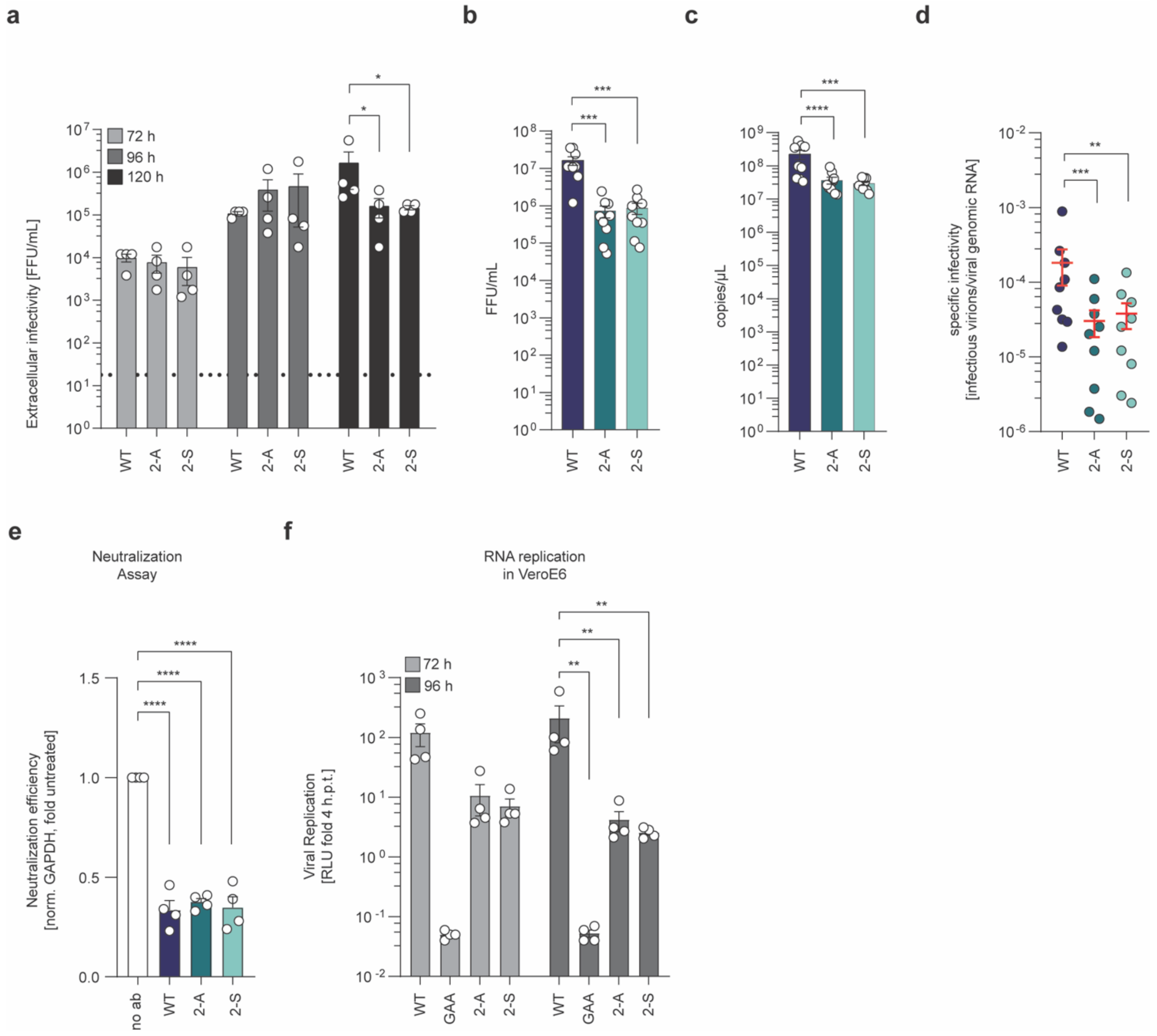

Supplementary Figure 6. Mutations affecting the CARC2 motif within the M-domain of the prM protein affect specific infectivity of ZIKV particles.
(a) Titers of infectious extracellular virus particles as determined by TCID50 assays. Huh7-Lunet cells were transfected with *in vitro* transcripts of synZIKV-H/PF/2013 and supernatants were collected at 72, 96 and 120 h.p.t. Data are mean ± SEM (n=4). Two-way ANOVA with Fisher's LSD test, $*P<0.05$. (WT vs 2-A, $P=0.0277$; WT vs 2-S, $P=0.0263$). **(b-d)** Supernatants of transfected Huh7-Lunet cells were collected 120 h.p.t. and concentrated by ultracentrifugation through a sucrose cushion. Virus titers were determined by TCID50 assays (b). Viral RNA contained in purified virus stocks was quantified by probe-based qRT-PCR (c). Specific infectivity (ratio of TCID50 value/viral genome RNA copy number) of WT and CARC2 mutants was calculated for each virus stock produced (d). Data are mean ± SEM. n=9 individual stock productions. Two-tailed ratio paired t test, $**P<0.01$, $***P<0.001$, $****P<0.0001$. ((b) WT vs 2-A, $P=0.0001$; WT vs 2-S, $P=0.0003$; (c) WT vs 2-S, $P=0.001$; (d) WT vs 2-A, $P=0.001$; WT vs 2-S, $P=0.0045$). **(e)** Neutralization assay. WT and CARC2 mutants (5,000 genome copies/cell) were incubated for 2 hours with the neutralizing E-specific antibody 4G2 at 37°C prior to inoculation of precooled VeroE6 cells. After 90 minutes total RNA was extracted, and virus RNA contained therein was quantified by qRT-

PCR (n=4). GAPDH was used for normalization. Relative neutralization of WT and CARC2 mutants is plotted after normalization to the non-treated (no ab) control. Data are mean ± SEM. One-way ANOVA with Dunnett's test, *****P*<0.0001. **(f)** VeroE6 cells were transfected with *in vitro* transcripts of synZIKV-R2A-H/PF/2013 and virus spread was indirectly assessed by monitoring *Renilla* luciferase activity at 72 and 96 h.p.t. Relative light units (RLU) normalized to the 4-hour value reflecting transfection efficiency are plotted. Data are mean ± SEM. n=4 independent experiments. Two-way ANOVA with Fisher's LSD test, ***P*<0.01. (WT vs GAA, *P*=0.0056; WT vs 2-A, *P*=0.0065; WT vs 2-S, *P*=0.0061).

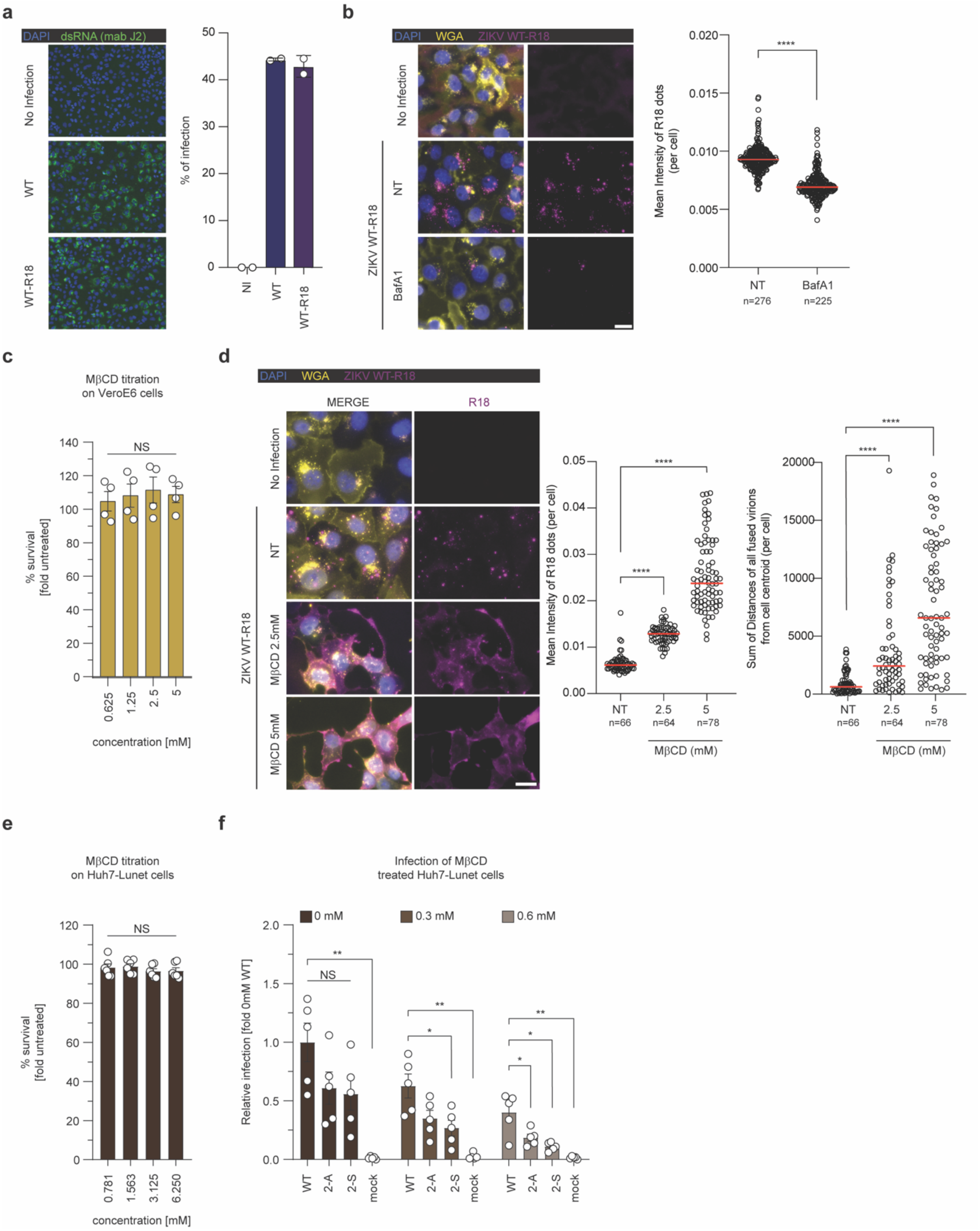

Supplementary Figure 7. ZIKV fusion is affected by cholesterol levels in host target membranes.

(a) Infectivity of Zika R18-labeled virions. VeroE6 cells were infected with ZIKV WT and WT-R18 (MOI=2). After 24 hours, infection was assessed by immunofluorescence microscopy using a double-stranded RNA specific antibody. Percentage of infection was estimated using the cell profile software package. Data are mean ± SD (n=2). Two-tailed Mann-Whitney test, ****P<0.0001. **(b, d)** ZIKV fusion is inhibited by bafilomycin A1 (b). Extraction of cellular cholesterol causes premature viral fusion (d).

VeroE6 cells were pretreated for 1 hour with either 100 nM bafilomycin A1 (b) or the cholesterol solubilizing drug methyl-β-cyclodextrin (MβCD) (d) followed by infection with WT-R18 (MOI=2). At 2 h.p.i., R18 signals were recorded by fluorescence microscopy. Nuclei were visualized using DAPI, plasma membranes were visualized using wheat germ agglutinin (WGA) conjugated with Alexa fluor 488. Scale bar: 20 μm. R18 signal intensities and distance to the cell centroid (nucleus) were quantified using the cell profiler software package ((b) n=2, (d) n=3). Two-tailed Mann-Whitney test, ****P<0.0001. **(c, e)** VeroE6 (n=4) (c) and Huh7-Lunet (n=6) (e) cells were treated with MβCD. After 24 hours cell viability was determined by measuring ATP levels. Untreated control cells were used for normalization. Data are mean ± SEM. One-way ANOVA with Tukey's test. NS not significant. **(f)** ZIKV CARC2 mutants are more sensitive to low cholesterol levels in Huh7-Lunet cells. Cells were treated 3 hours with MβCD prior to infection (MOI=2). At 24 h.p.i., cells were fixed and processed for immunofluorescence microscopy. Relative infection was calculated by determining the percentage of ZIKV positive cells observed for the mutants and normalization to the untreated control (n=5). Data are mean ± SEM. Two-way ANOVA with Fisher's LSD test. *P<0.05, **P<0.01. (0 mM WT vs mock, P=0.0039; 0.3 mM WT vs 2-S, P=0.0227; WT vs mock, P=0.004; 0.6 mM WT vs 2-A, P=0.0496; WT vs 2-S, P=0.0192; WT vs mock, P=0.0079). NS not significant.

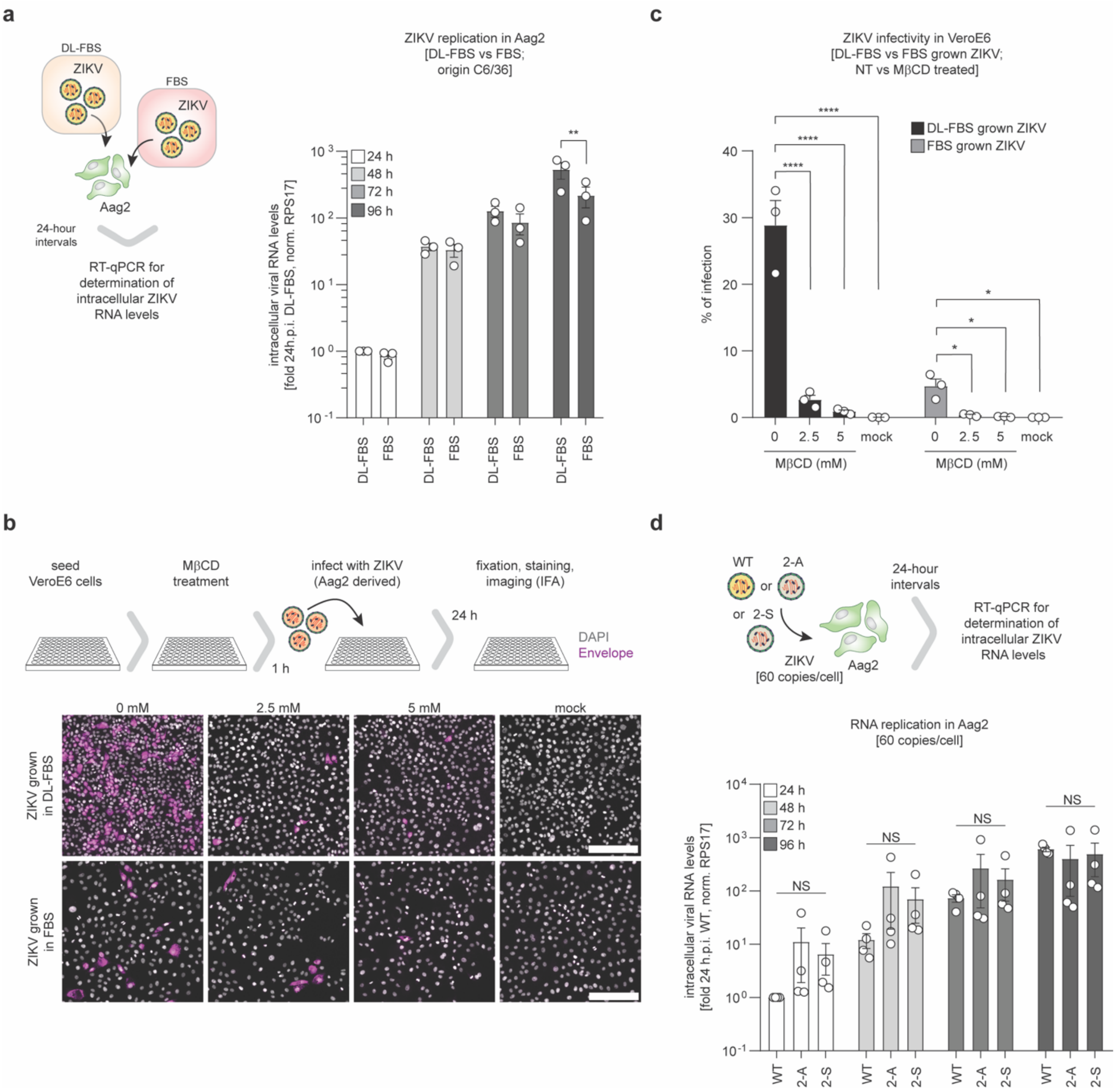

Supplementary Figure 8. Cholesterol is not a host dependency factor for ZIKV in mosquito cells.

(a) Experimental approach is shown on the left. Aag2 cells were infected with ZIKV H/PF/2013 (MOI=0.05 to allow extensive spread) in media supplemented with either delipidated FBS (DL-FBS) or lipidated (normal) FBS. ZIKV growth kinetics were assessed by quantifying intracellular viral RNA by qRT-PCR in 24-hour intervals over a period of 96 hours. For normalization, the ribosomal protein S17 (RPS17) was used. Relative replication is plotted after normalization to the 24-hour value (DL-FBS). n=3 independent experiments. Two-way ANOVA with Fisher's LSD test, ***P*<0.01. (DL-FBS vs FBS, *P*=0.0019). **(b)** Immunofluorescence microscopy to assess ZIKV infectivity in VeroE6 cells in the absence of cholesterol. VeroE6 cells were treated with MβCD for 1 hour prior to infection with ZIKV produced in Aag2 cells cultured in delipidated or normal FBS (cells in (a)). Infection was assessed 24 h.p.i. by detection of the E protein. Representative micrographs are shown (n=3). Scale bar: 20 μm. **(c)** Quantification of positive cells from (b). Data are mean ± SEM. n=3 independent experiments. Two-way ANOVA with Fisher's LSD test, **P*<0.05, *****P*<0.0001. (FBS: 0 vs 2.5, *P*=0.0353; 0 vs

5, *P*=0.027; 0 vs mock, *P*=0.0244). **(d)** Replication fitness of CARC2 mutants in Aag2 cells. Cells were seeded and infected with WT or CARC2 mutants using the same genome equivalence (60 copies/cell). Intracellular levels of ZIKV RNA were quantified by qRT-PCR. For normalization, RPS17 was used. Relative replication is plotted after normalization to the 24-hour value of WT (n=4). Data are mean ± SEM. Two-way ANOVA with Fisher's LSD test, NS not significant.

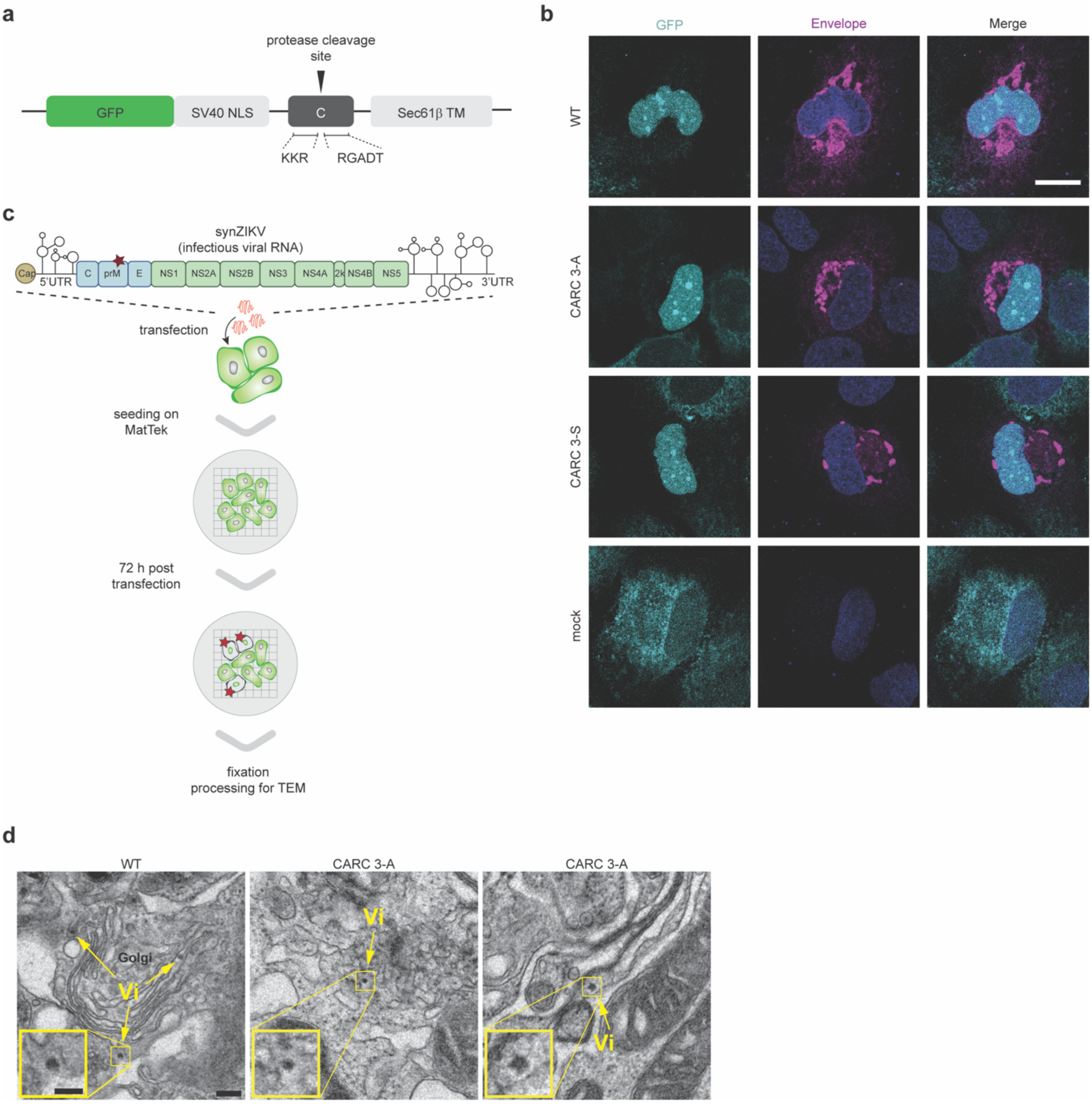

Supplementary Figure 9. A ZIKV reporter cell line suitable for correlative light and electron microscopy.
(a) Schematic of the ZIKV reporter construct stably expressed in target cells. The encoded fusion protein comprises GFP, the SV40 nuclear localization sequence (NLS), a linker containing the ZIKV protease cleavage site in capsid and the ER anchor sequence of the Sec61β transmembrane domain (TM). In infected cells, the linker is cleaved by the viral protease releasing GFP from the ER anchor to allow subsequent nuclear translocation of GFP via the SV40 NLS. **(b)** Representative images of Huh7-Lunet reporter cells transfected with synZIKV-H/PF/2013 *in vitro* transcripts. Positive cells were identified by nuclear GFP; ZIKV replication was verified by E-specific immunofluorescence. Scale bar: 20 μm. **(c)** Schematic representation of the experimental design. Huh7-Lunet cells stably expressing the ZIKV reporter were transfected with *in vitro* transcripts of WT synZIKV-H/PF/2013 or CARC3 mutants and seeded onto gridded MatTek dishes. At 72 h.p.t., positive cells were recorded, fixed, and processed for transmission electron microscopy (TEM). **(d)** Representative

TEM images of transfected cells are shown. Scale bar: 200 nm. Zooms of regions containing virions are shown in the inserts. Scale bar of inserts: 100 nm. Vi: virions.

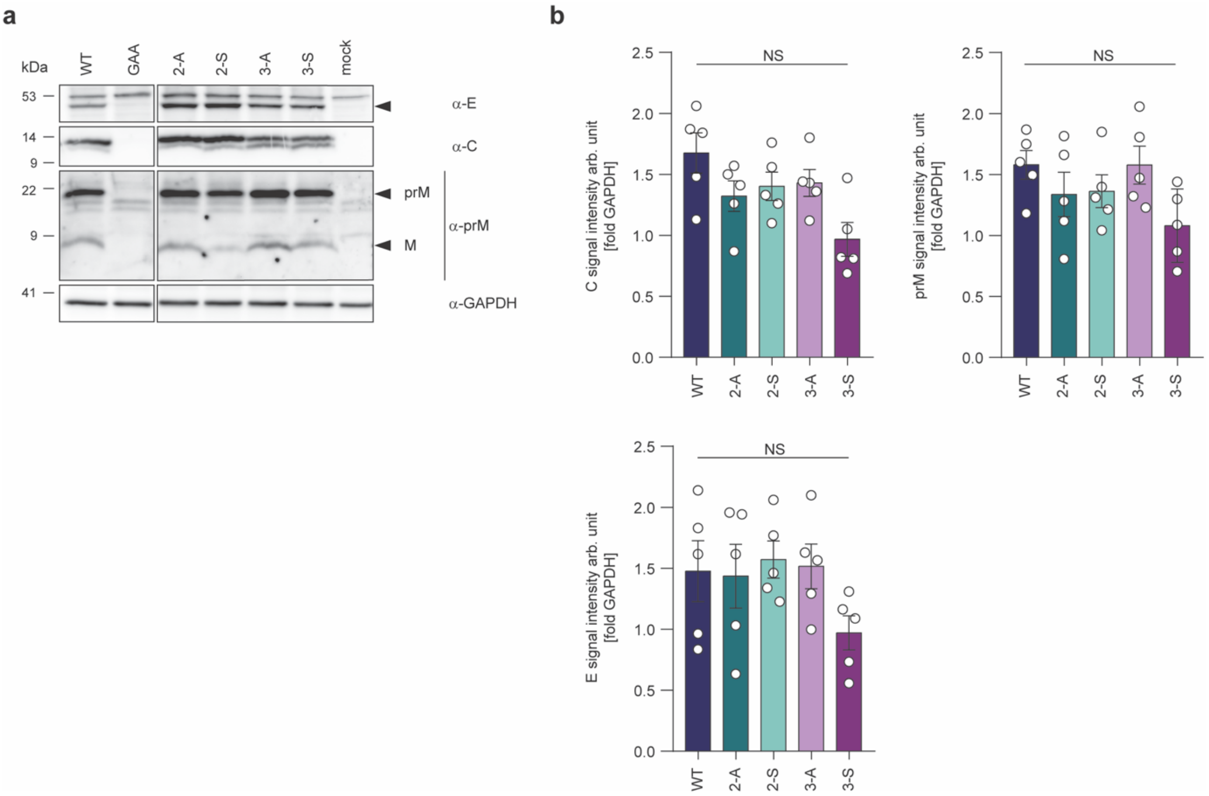

Supplementary Figure 10. Polyprotein processing.
(a) Huh7-Lunet cells were transfected with synZIKV-H/PF/2013 *in vitro* transcripts specified on the top and 48 hours later lysed and subjected to western blot analysis. Polyprotein processing was determined by detecting capsid, prM/M, and envelope. GAPDH was used as loading control. Molecular weights of proteins are indicated on the left (in kilodalton; kDa). **(b)** Relative abundance of polyprotein cleavage products was calculated by densitometry of western blots and normalizing signals of viral proteins in each sample to GAPDH loading control. n=5 independent experiments. Data are mean ± SEM. One-way ANOVA with Dunnett's test. NS not significant.

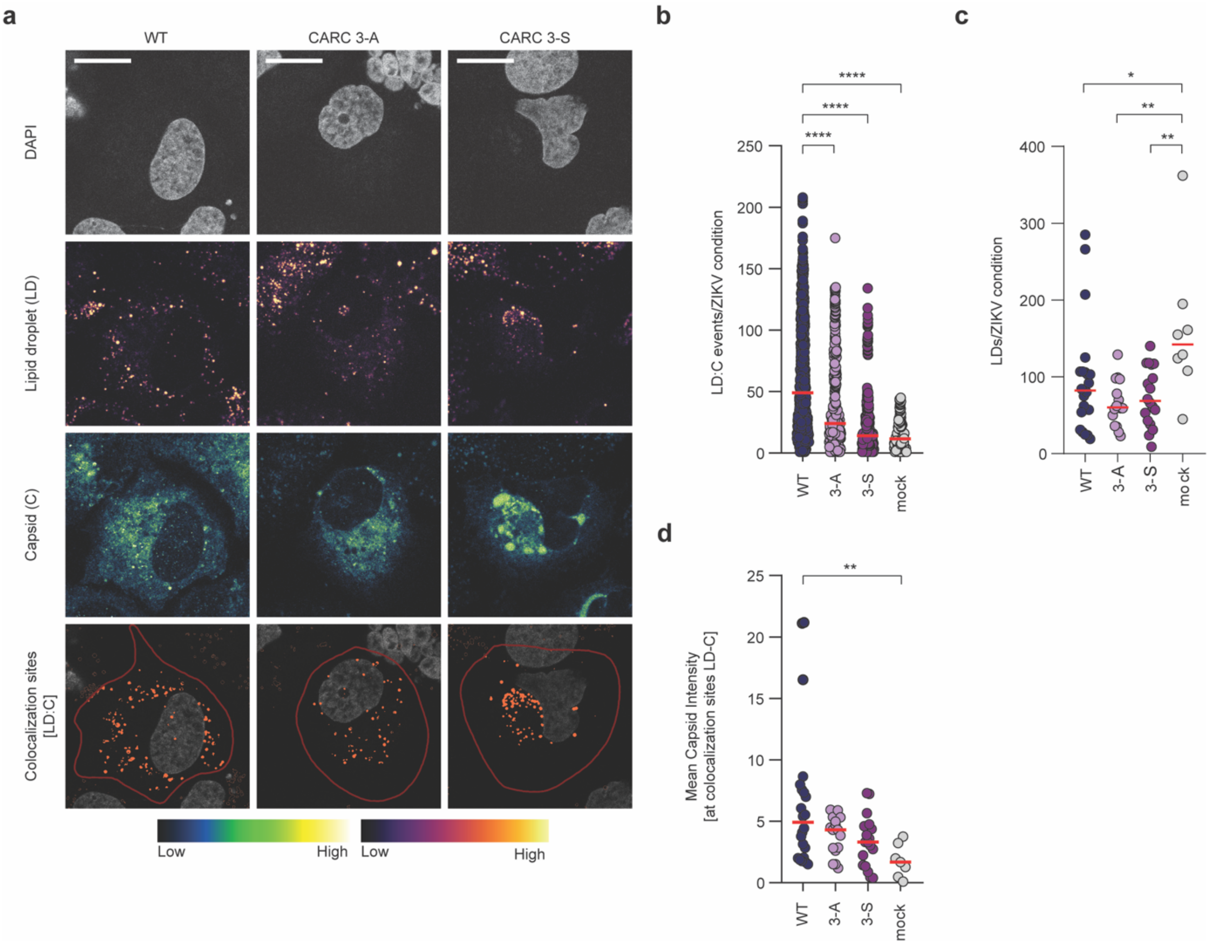

Supplementary Figure 11. Colocalization of capsid with lipid droplets.
(a) Huh7-Lunet cells were transfected with synZIKV-H/PF/2013 *in vitro* transcripts and at 48 h.p.t., cells were fixed and processed for immunofluorescence microscopy analysis (see Figure 7c). Signal intensities of lipid droplets (LDs) and capsid (C) are indicated on the bottom. Representative micrographs are shown (n=2). Scale bar: 20 μm. **(b, d)** Micrographs were analyzed using the cell profiler software package. Numbers of LD–C colocalization events + median (b) and mean intensity of capsid signals + median (d) are plotted. n=2 independent experiments. In total, 24 cells were analyzed for WT and 16 cells for each CARC3 mutant. Two-tailed Mann-Whitney test, $^{**}P<0.01$, $^{****}P<0.0001$. ((d) WT vs mock, $P=0.0036$). **(c)** Micrographs were analyzed using the FIJI software package. Numbers of lipid droplets + median per cell profile are plotted. n=2 independent experiments. Two-tailed Mann-Whitney test, $^{*}P<0.05$, $^{**}P<0.01$. (mock vs WT, $P=0.015$; mock vs 3-A, $P=0.0014$; mock vs 3-S, $P=0.0026$).

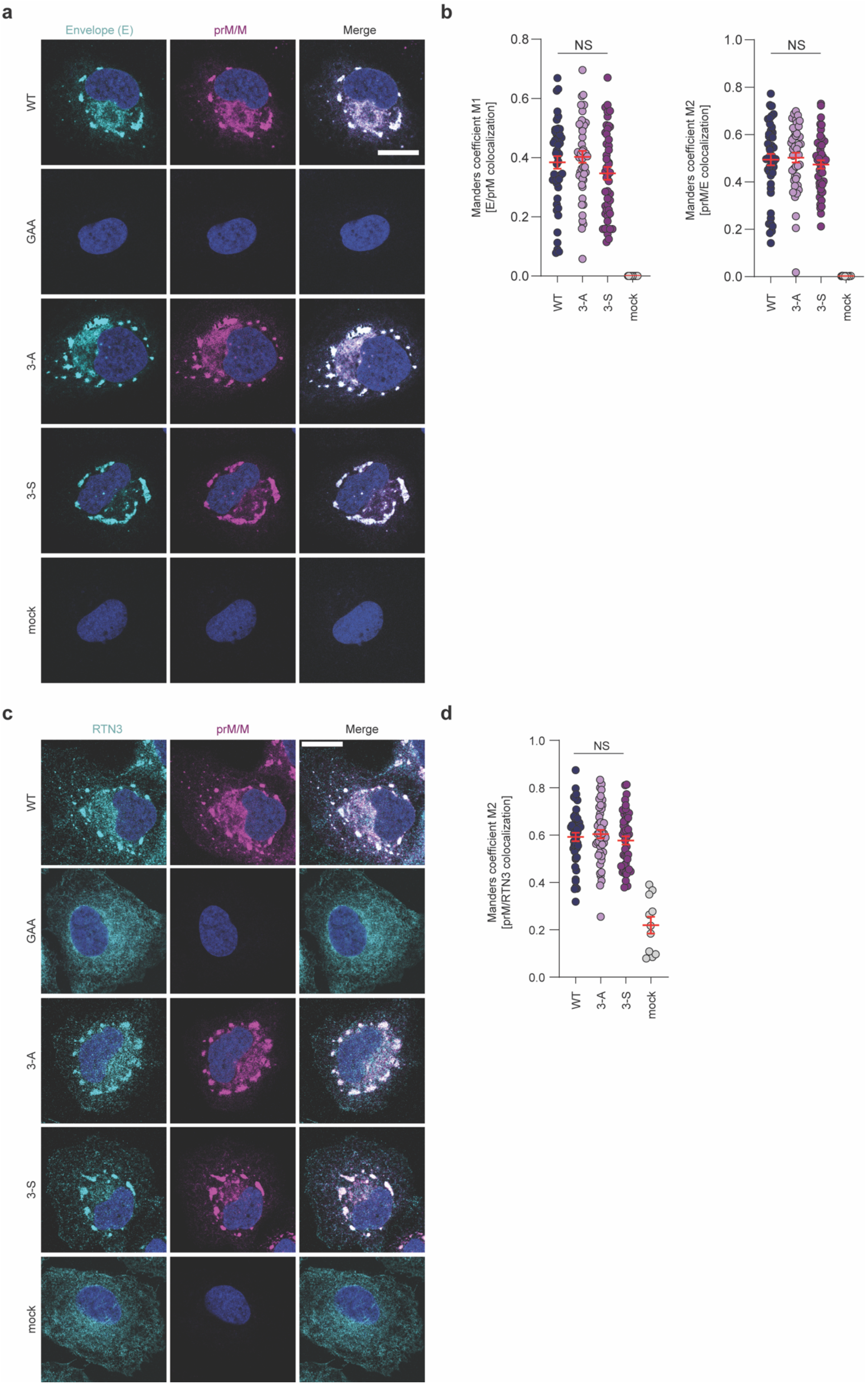
a
Envelope (E)
prM/M
Merge
WT
GAA
3-A
3-S
mock
b
NS
Manders coefficient M1 [E/prM colocalization]
Manders coefficient M2 [prM/E colocalization]
0.0
0.2
0.4
0.6
0.8
1.0
WT
3-A
3-S
mock
c
RTN3
prM/M
Merge
d
Manders coefficient M2 [prM/RTN3 colocalization]

Supplementary Figure 12. Subcellular localization of prM and E proteins
(a, c) Huh7-Lunet cells were transfected with synZIKV *in vitro* transcripts and seeded onto glass coverslips. After 48 hours, cells were PFA fixed and subjected to immunofluorescence analysis for prM (magenta) and envelope (cyan) (a) or prM (magenta) and RTN3 (cyan) (c). Scale bar: 20 μm. **(b, d)** Colocalization analysis of prM and E (b) or prM and RTN3 (d) in transfected cells. M1 (fraction of E overlapping with prM) and M2 (fraction of prM overlapping with E or RTN3) co-occurrence coefficients were estimated employing the Fiji JACoP plugin. Data are mean ± SEM derived from 46 analyzed cells. n=3 independent experiments. Kruskal-Wallis with Dunn's test. NS not significant.

Supplementary Tables

Supplementary Table 1 | Primers used for cloning.

Name	Sequence (5’-3’)
prM_full-length_Fw	GAAGCGAAAGCTAGCAACAGTATCAACAG
prM_full-length_Rev	CATTTTCTTGGAGCATGCAAACTTAGCGC
prM_R238L+Y240A +R246L_Fw	GAATCACTAGAAGCAACAAAGCACTTGATTCTAGTCGAAA
prM_R238L+Y240A +R246L_Rev	TTTCGACTAGAATCAAGTGCTTTGTTGCTTCTAGTGATTC
prM_R238L+Y240S +R246L_Fw	GAATCACTAGAAAGCACAAAGCACTTGATTCTAGTCGAAA
prM_R238L+Y240S +R246L_Rev	TTTCGACTAGAATCAAGTGCTTTGTGCTTTCTAGTGATTC
prM_R253L+F257A _Fw	TATTCCTAAACCCTGGCGCAGCGTTA
prM_R253L+F257A _Rev	TAACGCTGCGCCAGGGTTTAGGAATA
prM_R253L+F257S _Fw	TATTCCTAAACCCTGGCAGCGCGTTA
prM_R253L+F257S _Rev	TAACGCGCTGCCAGGGTTTAGGAATA
prM_K275L+Y278A _Fw	AACGAGCCAACTAGTCATAGCATTGGTC
prM_K275L+Y278A _Rev	GACCAATGCTATGACTAGTTGGCTCGTT
prM_K275L+Y278S _Fw	AACGAGCCAACTAGTCATAAGCTTGGTC
prM_K275L+Y278S _Rev	GACCAAGCTTATGACTAGTTGGCTCGTT
pTM_prM-HA_Fw	AAAACCATGGGAGCAGATACTAGTGTCGG
pTM_prM-HA_Rev	AAAAGGATCCCTAAGCGTAATCTGGAACATCGTATGGGTATGATCCTT GGTATTGCGGGGCAATCAGCAGTATCATG
pTM_Env_Fw	AAAACCATGGTCATCTACTTGGTCATGATACTGCTGATTGC
pTM_Env-Rev	AAAAGGATCCTTAAGCAGAGACAGCTGTGG
ZIKV_VLP_Fw	AAAACCATGGGAGCAGATACTAGTGTCGGAATTGTTGGCC
ZIKV_VLP_Rev	TTTTGGATCCTTAAGCAGAGACAGCTGTGGATAAGAAGATC
pTM_prM-Env-HA_Fw	AAAAAGGATCCATGAGTGTCGGAATTGTTGGCCTCCT

pTM_prM-Env_Rev	AAAAAGAATTCTTAAGCAGAGACAGCTGTGGATAAGAAGATC
pTM_prM-Env-HA_Rev	AAAAAGAATTCTTAAGCGTAATCTGGAACATCGTATGGGTATG ATCCAGCAGAGACAGCTGTGGATAAGAAGATC

Supplementary Table 2 | Antibodies used in this study.

Primary antibodies		
**Target**	**Company**	**Catalog number**
ZIKV capsid (IF: 1:250; WB: 1:1000)	Genetex	GTX133317
ZIKV prM (IF: 1:250; WB: 1:1000)	Genetex	GTX133584
ZIKV envelope (WB: 1:1000)	Genetex	GTX133314
HA (WB: 1:1000)	Invitrogen	PA1-985
GAPDH (WB: 1:1000)	Santa Cruz	Sc-365062
RTN3 (IF: 1:200)	Santa Cruz	Sc-374599
PanFlavi envelope (IF: 1:2; TCID50: 1:2)	ATCC	D1-4G2-4-15, HB-112
dsRNA J2 (IF: 1:250)	SCICONS	10010500
**Secondary antibodies**		
Goat anti-rabbit IgG HRP (WB: 1:5000)	Sigma Aldrich	A6154
Goat anti-mouse IgG HRP (WB: 1:10000; TCID50: 1:500)	Sigma Aldrich	A4416
Alexa Fluor 488 donkey anti-mouse IgG (IF: 1:1000)	ThermoFisher	A-21131
Alexa Fluor 568 donkey anti-rabbit IgG (IF: 1:1000)	ThermoFisher	A-10042
Alexa Fluor 647 donkey anti-mouse IgG (IF: 1:500)	ThermoFisher	A-32787
**Probes**		
BODIPY	Invitrogen	D3922
Octadecyl rhodamine (R18)	Sigma Aldrich	83685
WGA-Alexa 488 conjugated	ThermoFisher	W11261

Supplementary Table 3| Primers and probes used for qPCR.

qPCR primers		
**Name**	**Forward primer (5'-3')**	**Reverse primer (5'-3')**
GAPDH	GAAGGTGAAGGTCGGAGTC	GAAGATGGTGATGGGATTTC
ZIKV	ACTCAACGCAATCCTGGAAG	AAGTACGATTTCCCCCAAGC
RPS17	CACTCCGAGGTCCGTGGTAT	GGACACTTCGGGCACGTAGT
**qPCR probes**		
**Name**	**Sequence (5'-3')**	
GAPDH	Cy5 - CAAGCTTCCCGTTCTCAGCCT - BHQ3	
ZIKV	FAM - TGGAGTTCAACTGACGGTCGTTGTG - BHQ1	
**RT-PCR primers**		
**Name**	**Forward primer (5'-3')**	**Reverse primer (5'-3')**
Envelope		CACATCAGCAGAGACAGCTGT
Structural genes	TCATGGGCCCATCAGGATGG	CTTGTCAAGGTAGGCTTCACCTTGT
