# Supplementary material for: Zika virus prM protein contains cholesterol binding motifs required for virus entry and assembly
Source: Nat Commun. 2023 Nov 13;14:7344. doi: 10.1038/s41467-023-42985-x (PMC10643666; doi:10.1038/s41467-023-42985-x)
Supplement: Supplementary file 3 — Reporting Summary [file 41467_2023_42985_MOESM3_ESM.pdf]

nature portfolio

Corresponding author(s): Ralf Bartenschlager

Last updated by author(s): 15-October-2023

Reporting Summary

Nature Portfolio wishes to improve the reproducibility of the work that we publish. This form provides structure for consistency and transparency in reporting. For further information on Nature Portfolio policies, see our Editorial Policies and the Editorial Policy Checklist.

Statistics

For all statistical analyses, confirm that the following items are present in the figure legend, table legend, main text, or Methods section.

n/a	Confirmed	
☐	☒	The exact sample size (*n*) for each experimental group/condition, given as a discrete number and unit of measurement
☐	☒	A statement on whether measurements were taken from distinct samples or whether the same sample was measured repeatedly
☐	☒	The statistical test(s) used AND whether they are one- or two-sided<br>*Only common tests should be described solely by name; describe more complex techniques in the Methods section.*
☒	☐	A description of all covariates tested
☐	☒	A description of any assumptions or corrections, such as tests of normality and adjustment for multiple comparisons
☐	☒	A full description of the statistical parameters including central tendency (e.g. means) or other basic estimates (e.g. regression coefficient) AND variation (e.g. standard deviation) or associated estimates of uncertainty (e.g. confidence intervals)
☐	☒	For null hypothesis testing, the test statistic (e.g. *F*, *t*, *r*) with confidence intervals, effect sizes, degrees of freedom and *P* value noted<br>*Give P values as exact values whenever suitable.*
☒	☐	For Bayesian analysis, information on the choice of priors and Markov chain Monte Carlo settings
☒	☐	For hierarchical and complex designs, identification of the appropriate level for tests and full reporting of outcomes
☒	☐	Estimates of effect sizes (e.g. Cohen's *d*, Pearson's *r*), indicating how they were calculated

Our web collection on statistics for biologists contains articles on many of the points above.

Software and code

Policy information about availability of computer code

Data collection	- Immunofluorescence image data collection was performed using either the Zeiss Cell Discoverer7, Leica SP8 microscope, or Nikon Eclipse Ti microscope, Images were analyzed either using the Cell Profiler software or FIJI software<br>- Western blot data collection was performed using the INTAS ECL chemocam imager (ChemoStar software package)<br>- EM data collection was performed using the JEOL JEM-1400 transmission electron microscope (image analysis was performed using the FIJI software)<br>- qPCR data collection was performed using the CFX96 Real-Time System from BioRad, Analysis was done using the BioRad CFX software<br>- Renilla luciferase data collection was performed using the Tube Luminometer (lumat LB) from Berthold Technologies Bioanalytics<br>- All MD simulations were performed using GROMACS 2021.
Data analysis	- all image based segmentation was performed in the Cell Profiler software 3.0, except Supplementary Figure 1, 2, and 12, which were analyzed with the FIJI software package<br>- qPCR data were analyzed using the CFX Maestro V2 program from BioRad<br>- GraphPad Prism software (version 8) was used for plotting the results and performing the statistical tests<br>- Cell Profiler software 3.0 was used for analyze the colocalization events and capsid intensities (Figure 7 and Supplementary Figure 11) as defined in the figure legends<br>- Western blots were analyzed using the ImageLab1D (INTAS) software package, processing of western blots for representation in the figures was done using the FIJI software<br>- Visual Molecular Dynamics (VMD) and python packages scikit-learn and MDTraj were used for visualization and analyses.

For manuscripts utilizing custom algorithms or software that are central to the research but not yet described in published literature, software must be made available to editors and reviewers. We strongly encourage code deposition in a community repository (e.g. GitHub). See the Nature Portfolio guidelines for submitting code & software for further information.

Data

Policy information about availability of data

All manuscripts must include a data availability statement. This statement should provide the following information, where applicable:

- Accession codes, unique identifiers, or web links for publicly available datasets
- A description of any restrictions on data availability
- For clinical datasets or third party data, please ensure that the statement adheres to our policy

The source data underlying Figs. 1c-e, 2d-e, 3e-f, 4b-c, e, 5a, c-e, g, 6a-d, f-g, 7a-b, d-h, and Supplementary Figs. 1c, 2b, d, 3a-b, 4a-c, 5b-d, 6a-h, 7a-c, 9a-b, 10b-c, 11b, d are provided in the Source Data file. The MD simulations data generated in this study have been deposited online and can be accessed via doi: 10.5281/zenodo.8268517. The published article includes all datasets generated or analyzed during this study.
The protein structure used for MD simulations (PDB ID:5IRE) can be accessed via https://doi.org/10.2210/pdb5IRE/pdb

Research involving human participants, their data, or biological material

Policy information about studies with human participants or human data. See also policy information about sex, gender (identity/presentation), and sexual orientation and race, ethnicity and racism.

Reporting on sex and gender	N/A
Reporting on race, ethnicity, or other socially relevant groupings	N/A
Population characteristics	N/A
Recruitment	N/A
Ethics oversight	N/A

Note that full information on the approval of the study protocol must also be provided in the manuscript.

Field-specific reporting

Please select the one below that is the best fit for your research. If you are not sure, read the appropriate sections before making your selection.

☒ Life sciences ☐ Behavioural & social sciences ☐ Ecological, evolutionary & environmental sciences

For a reference copy of the document with all sections, see nature.com/documents/nr-reporting-summary-flat.pdf

Life sciences study design

All studies must disclose on these points even when the disclosure is negative.

Sample size	- sample size was not pre-determined<br>- in our opinion, having experiments performed with 10,000-250,000 cells in infection and transfection assays are sufficient and reliable to predict an associated outcome.<br>- no statistical methods were used to predetermine sample sizes<br>- Sample sizes are stated in the figure legends when applicable
Data exclusions	- No data was excluded in any of the experiments. For image analysis random sampling was done to keep an even number of cells analyzed between cells. For the EM data analysis more cells were analyzed for the mutants due to the reduced number of virions formed.
Replication	- Experiments were biologically and experimentally reproduced on separate days, separate batches of plasmid preparations and in vitro transcripts, separate batches of viruses, different passages of the cell lines to confirm replication of the effect. The exact number of biological repetitions are stated in the figure legends for each figure subsection
Randomization	- No randomization methods were used as covariants are not relevant to our study. Random sampling for IF images investigating the colocalization events was done to keep sample size constant among the different conditions. The same number of cells were used to determine the statistical significance. this applies to Supplementary Figure 1, 2, and 12.
Blinding	- Blinding was not done during data acquisitions or analysis. Infection assays were high-throughput and analyses were done in an automated manner. Analysis using softwares minimized the occurrence of any bias.

Reporting for specific materials, systems and methods

We require information from authors about some types of materials, experimental systems and methods used in many studies. Here, indicate whether each material, system or method listed is relevant to your study. If you are not sure if a list item applies to your research, read the appropriate section before selecting a response.

Materials & experimental systems

n/a	Involved in the study
☐	☒ Antibodies
☐	☒ Eukaryotic cell lines
☒	☐ Palaeontology and archaeology
☒	☐ Animals and other organisms
☒	☐ Clinical data
☒	☐ Dual use research of concern
☒	☐ Plants

Methods

n/a	Involved in the study
☒	☐ ChIP-seq
☒	☐ Flow cytometry
☒	☐ MRI-based neuroimaging

Antibodies

Antibodies used

Primary antibodies:
- ZIKV capsid (Genetex, GTX133317) dilution for western blot: 1:1000; dilution in IF: 1:250
- ZIKV prM (Genetex, GTX133584) dilution for western blot: 1:1000; dilution in IF: 1:250
- ZIKV envelope (Genetex, GTX133314) dilution for western blot: 1:1000
- PanFlavi Envelope (ATCC, D1-4G2-4-15, HB-112) dilution for TCID50 and IF: 1:2 (this antibody was produced in house by collecting the supernatant containing the secreted antibodies)
- HA (Invitrogen, PA1-985) dilution for western blot: 1:1000
- GAPDH (Santa-Cruz, Sc-365062) dilution for western blot: 1:1000
- RTN3 (Santa Cruz, Sc-374599) dilution for IF: 1:200
- dsRNA (SCICONS, 10010500) dilution for IF: 1:250

secondary antibodies
- goat anti-rabbit IgG HRP (Sigma Aldrich, A6154) dilution for western blot: 1:5000
- goat anti-mouse IgG HRP (Sigma Aldrich, A4416) dilution for western blot: 1:10000; dilution for TCID50: 1:500
- Alexa Fluor 488 donkey anti-mouse IgG (ThermoFisher. A-21131) dilution for IF: 1:1000
- Alexa Fluor 568 donkey anti-rabbit IgG (ThermoFisher, A-10042) dilution for IF: 1:1000
- Alexa Fluor 647 donkey anti-mouse IgG (ThermoFisher, A-32787) dilution for IF: 1:500

All antibodies, except for the panFlavi Env antibody, are commercially available. panEnv antibody was produced in house. Details are provided in the Supplementary Information file.

Validation

- primary antibodies were initially validated by comparing ZIKV infected cells with mock infected cells. For this samples were generated to be subjected either for western blot or IF analysis.
- The panFlavi Envelope antibody is used in the Molecular Virology Department, Heidelberg, to analyze TCID50 for dengue virus.
- All antibodies were initially tested by the recommended dilutions given by the supplier. To ensure the specificity for ZIKV proteins, mock cells (no infection) were analyzed in parallel.
- Given the information by the supplier for the molecular weight of the protein, western blots were validated by checking for the protein at the expected size.
- The HA antibody was analyzed and validated by including untagged samples in the immunoprecipitation experiment.
- Subcellular localization of the viral proteins was confirmed by costaining cellular organelles with respective antibodies (i.e., RTN3).
- Throughout this study, as controls the replication deficient mutant and uninfected (mock) cells were processed in parallel to the ZIKV infected/transfeted samples to ensure the specificity of the antibody.

Eukaryotic cell lines

Policy information about cell lines and Sex and Gender in Research

Cell line source(s)

- Huh7-Lunet cells (Friebe et al, 2005; doi: 10.1128/JVI.79.1.380-392.2005)
- Huh7-Lunet T7 cells (Apple et al, 2005; doi: 10.1128/JVI.79.5.3187-3194.2005)
- HEK 293T (ATCC CRL-3216)
- VeroE6 (ATCC CRL-1586)
- A549 (ATCC CCL-185)
- Huh7-Lunet ZIKV reporter cell line (generated in this study)
- C6/36 (ECACC 89051705)
- Aag2 (provided by Alain Kohl)
- JEG3 (provided by Udo Markert)

Authentication

- A549 ATCC cells obtained from ATCC were authenticated by cell-typing performed by Microsynth AG, Switzerland.
- Remaining cell lines were not authenticated

Mycoplasma contamination	- All cell lines have been checked and tested negative for mycoplasma contamination
Commonly misidentified lines (See ICLAC register)	- no cell lines from the ICLAC register
