# Supplementary material for: Zika virus prM protein contains cholesterol binding motifs required for virus entry and assembly
Source: Nat Commun. 2023 Nov 13;14:7344. doi: 10.1038/s41467-023-42985-x (PMC10643666; doi:10.1038/s41467-023-42985-x)
Supplement: Supplementary file 4 — Description of Additional Supplementary Files [file 41467_2023_42985_MOESM4_ESM.pdf]

Description of additional supplementary files

Title: Supplementary Movie 1

Description:

LDA analysis was conducted on the WT M-protein using cholesterol concentrations as distinct classes and heavy atom cartesian coordinates as the feature vector. The video shows the interpolation between the extrema of the feature vector's projection onto the first LDA component. This component effectively distinguishes between the different classes. Notably, it captures the narrowing and widening of the transmembrane helices in response to varying cholesterol concentrations.
